# Prolonged activation of S6K1 does not suppress IRS or PI-3 kinase signaling during muscle cell differentiation

**DOI:** 10.1186/1471-2121-11-37

**Published:** 2010-05-27

**Authors:** D Lee Hamilton, Andrew Philp, Matthew G MacKenzie, Keith Baar

**Affiliations:** 1 Division of Molecular Physiology, James Black Centre, University of Dundee, Dundee, UK

## Abstract

**Background:**

Myogenesis in C2C12 cells requires the activation of the PI3K/mTOR signaling pathways. Since mTOR signaling can feedback through S6K1 to inhibit the activation of PI3K, the aim of this work was to assess whether feedback from S6K1 played a role in myogenesis and determine whether siRNA mediated knockdown of S6K1 would lead to an increased rate of myotube formation.

**Results:**

S6K1 activity increased in a linear fashion following plating and was more than 3-fold higher after Day 3 of differentiation (subconfluent = 11.09 ± 3.05, Day 3 = 29.34 ± 3.58). IRS-1 levels tended to increase upon serum withdrawal but decreased approximately 2-fold (subconfluent = 0.88 ± 0.10, Day 3 = 0.42 ± 0.06) 3 days following differentiation whereas IRS-2 protein remained stable. IRS-1 associated p85 was significantly reduced upon serum withdrawal (subconfluent = 0.86 ± 0.07, Day 0 = 0.31 ± 0.05), remaining low through day 1. IRS-2 associated p85 decreased following serum withdrawal (subconfluent = 0.96 ± 0.05, Day 1 = 0.56 ± 0.08) and remained suppressed up to Day 3 following differentiation (0.56 ± 0.05). Phospho-tyrosine associated p85 increased significantly from subconfluent to Day 0 and remained elevated throughout differentiation. siRNA directed against S6K1 and S6K2 did not result in changes in IRS-1 levels after either 48 or 96 hrs. Furthermore, neither 48 nor 96 hrs of S6K1 knockdown caused a change in myotube formation.

**Conclusions:**

Even though S6K1 activity increases throughout muscle cell differentiation and IRS-1 levels decrease over this period, siRNA suggests that S6K1 is not mediating the decrease in IRS-1. The decrease in IRS-1/2 associated p85 together with the increase in phospho-tyrosine associated p85 suggests that PI3K associates primarily with scaffolds other than IRS-1/2 during muscle cell differentiation.

## Background

During development, multinucleated muscle fibers form from the terminal differentiation and fusion of individual myogenic progenitors, or myoblasts [1]. This process is recapitulated during the process of skeletal muscle regeneration in response to trauma, disease, or contraction mediated injury [2]. As a result of the importance of this process, the regulation of myoblast differentiation and fusion has been widely studied in tissue culture using both primary and transformed myoblasts. In 2-dimensional cell culture, myoblasts are maintained in a proliferative state by providing media with high serum content and maintaining a relatively low confluence. Growing the myoblasts to full confluence or changing to a low serum growth media induces terminal differentiation. The combination of high confluence and low serum growth media induces both myoblast differentiation and fusion, resulting in the formation of myotubes.

Myogenesis is regulated at multiple levels. The degree of confluence and the level of growth factors within the media are transduced by a series of kinase signaling pathways [3]. How myoblasts signal confluence is currently unclear. At high confluence, cadherins are thought to signal to p38 MAPK either through a Rho kinase (ROCK) [4] or Cdo/JLP dependent [5] mechanism. The activation of p38 enhances the transcriptional activity of several MRFs [6] and in this way may initiate myoblast differentiation.

Growth factor regulation of myogenesis is better understood. When growth factors are low, myoblasts begin to secrete IGF-II, which is required for, and determines, the rate of differentiation [7]. IGF-II is thought to initiate an autocrine signaling cascade through the IGF-I receptor [8] and the insulin receptor substrates (IRS) 1 and 2, that activates MAPK and PI3K (phosphoinositide-3 kinase) [9]. PI3K activates PKB (protein kinase B/akt), and either PI3K or PKB is sufficient for myoblast differentiation and fusion [10]. PI3K and PKB drive differentiation by inhibiting GSK3 [11], increasing the transcriptional activity of MyoD [12], and activating the mammalian target of rapamycin (mTOR; [13]. mTOR expression and activity increase during differentiation [14] leading to an increase in the activity of its downstream target, S6K1 [14,15]. However, neither the kinase activity of mTOR nor S6K1 are required for differentiation [16]. Even though S6K1 activity is not required for muscle cell differentiation, S6K1 can regulate the activation of PKB by IGF-II through IRS-1 protein, mRNA, and activity [17-19]. Where S6K1 is constitutively active, as in TSC2^-/- ^cells, IRS-1 function is potently suppressed [17].

Considering the important role of the IGF-II signaling through IRS-1 to PKB in the control of differentiation, and the ability of S6K1 to block IRS-1 signaling, it seems paradoxical that S6K1 would be activated during differentiation. The aim of the current work was to assess whether the negative feedback between S6K1 and IRS-1 was maintained during myogenesis. We hypothesised that the activation of S6K1 during differentiation would correlate with a reduction in IRS-1 function and that inhibiting S6K1 activation would increase myotube formation.

## Results

### Myotube formation and muscle specific gene expression

**Figure 1:**
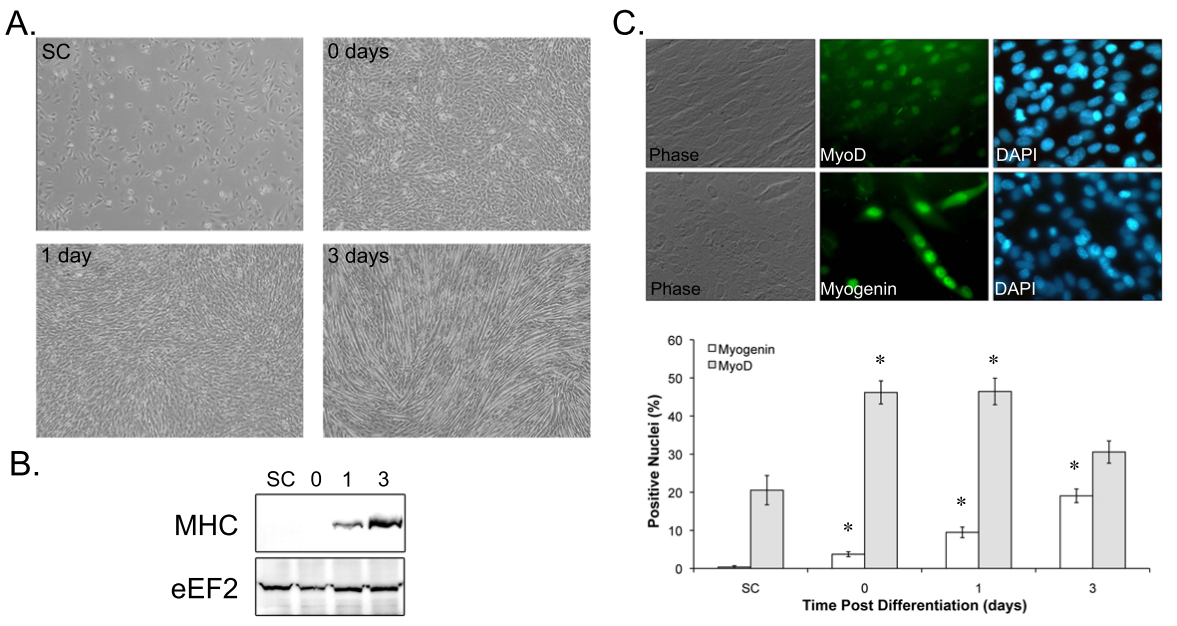
**Myotube formation and muscle specific gene expression**. A) Representative phase contrast images of C2C12 cells 3hrs post plating (subconfluent; SC), 24 h post plating (Day 0), 24 h after achieving confluence (Day 1), or 72 hrs following differentiation (Day 3). B) Myosin heavy chain protein content at subconfluent (SC), 24 h post plating (Day 0), 24 h after achieving confluence (Day 1), or 72 hrs following differentiation (Day 3). C) Representative images of MyoD and myogenin immunohistochemistry and the quantification of positive nuclei. * indicates significantly different from subconfluent (SC) values.

Three hours after plating, the subconfluent cells attached and spread to reach ~70% confluence (Figure 1). At Day 0, the cells formed a confluent monolayer. At Day 1, the cells began to align and fuse and expressed low levels of myosin heavy chain (Figure 1B). By Day 3 large numbers of myotubes and high myosin heavy chain levels were evident. These time points were selected since the subconfluent to Day 0 interval could be used to determine contact-dependent events, the Day 0 to Day 1 interval could be used to identify growth factor-dependent differentiation events, and the 3 day point could be used to determine the effects of an intervention on fusion.

The percentage of nuclei stained with MyoD or myogenin and the levels of myosin heavy chain were assessed to give a measure of the stage of myogenesis. Nuclei stained with MyoD increased approximately 2-fold upon reaching confluence (Figure 1C; subconfluent = 22 ± 7.3%; versus Day 0 = 47 ± 5.1%), remained elevated once growth factors were removed for 24 hrs (Day 1 = 46.8 ± 6.4%), and returned towards subconfluent levels following another 48 hrs in DM (Day 3 = 31 ± 4.1%). Myogenin expression increased progressively following plating, peaking 72 hrs following serum withdrawal (Figure 1C).

### Differentiation induced signaling

**Figure 2:**
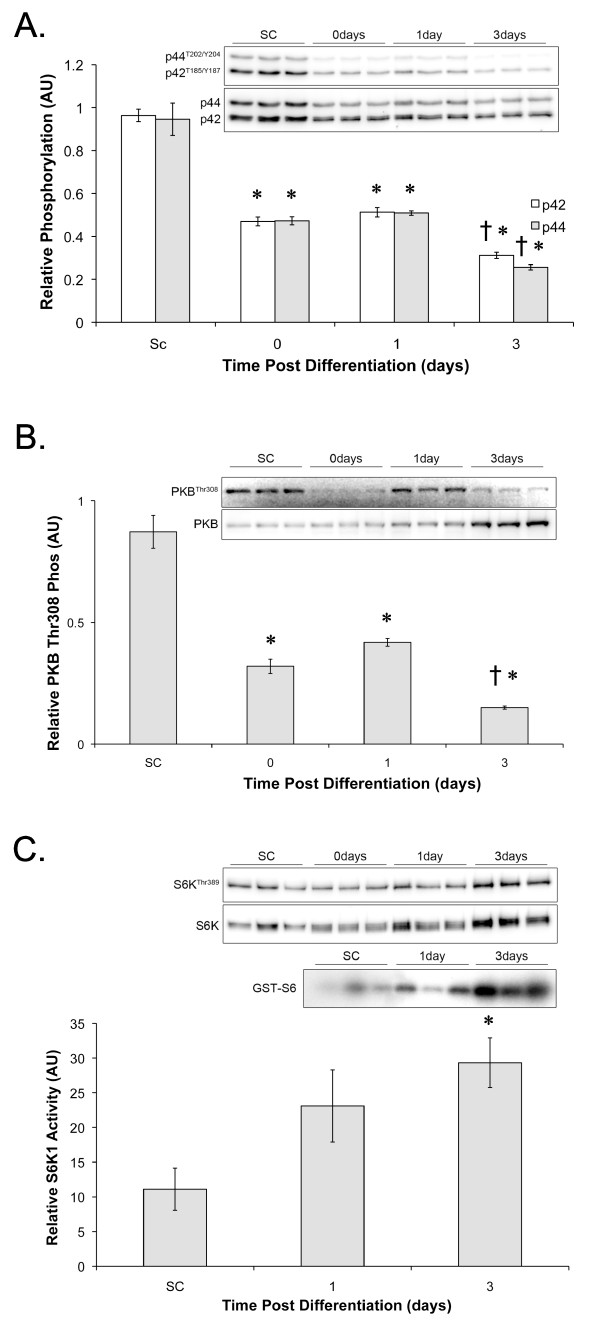
**Differentiation decreases p42/44 and PKB signaling whereas S6K activity increases**. A) p44/42 MAPK Thr202/Tyr204 phosphorylation relative to total p44/42 (p-p44/42), B) PKB Thr308 phosphorylation relative to total PKB (p-PKB) and C) S6K1 phosphorylation and activity determined relative to total S6K1 3 hrs post plating (subconfluent; SC), 24 h post plating (Day 0), 24 h after achieving confluence (Day 1), or 72 hrs following differentiation (Day 3). * indicates significantly different from SC, † indicates significantly different from Day 1. Values were obtained by normalising specific phosphorylation to total protein content as described in methods.

To begin to understand the dynamics of the signaling events that may lead to differentiation, the phosphorylation of p44/42 at Thr202/Tyr204 and PKB at Thr308, and the phosphorylation and activity of S6K1 were determined at the various stages of differentiation. The phosphorylation of p44/42 decreased significantly upon reaching confluence and remained significantly suppressed up to 72 hrs following serum withdrawal (Figure 2A). Phosphorylation of PKB on the other hand appears to have a bi-phasic response. Upon reaching confluence, PKB phosphorylation decreased approximately 3-fold (SC = 0.87 ± 0.07, Day0 = 0.32 ± 0.02). From Day 0 to Day 1, PKB phosphorylation and total protein increased marginally (Day0 = 0.32 ± 0.02, Day 1 = 0.42 ± 0.02), and then from Day 1 to Day 3, PKB phosphorylation decreased and the total protein increased resulting in a large decrease in the ratio of phospho- to total PKB (Day 3 = 0.15 ± 0.01; Figure 2B). S6K1 phosphorylation and activity increased approximately 3-fold from SC (11.10 ± 3.05) to Day 3 (29.33 ± 3.57; Figure 2C).

### IRS-1/2 Expression and associated p85

**Figure 3:**
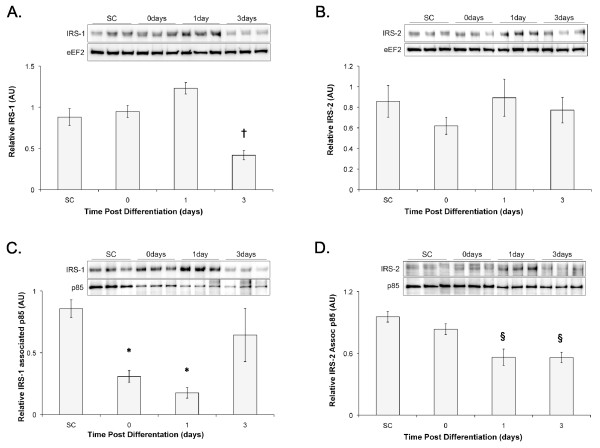
**IRS-1/2 expression and associated p85 decrease throughout differentiation**. A) IRS-1 and B) IRS-2 total protein was measured via western blots and normalised to relative eEF2 protein content and C) IRS-1 and D) IRS-2 associated p85 was determined as described in methods. All measures were made at 3 (subconfluent; SC), 24 (Day 0), 48 (Day 1), or 96 hours (Day 3) following plating. * indicates significantly different from SC, † indicates significantly different from all other time points, § indicates significantly different from SC and Day 0.

To better understand the role that IRS-1 and IRS-2 play in signaling during myogenesis, IRS1/2 protein level and PI3K association were determined. IRS-2 protein (Figure 3B) remains stable throughout the time course, whereas IRS-1 protein tended to increase to Day 1 before reducing approximately 2-fold by Day 3 (subconfluent = 0.88 ± 0.10, Day 3 = 0.42 ± 0.06; Figure 3A). The association of IRS-1 with p85 significantly reduced upon serum withdrawal (subconfluent = 0.86 ± 0.07, Day 0 = 0.31 ± 0.05). Even though the absolute amount of p85 precipitated remain the same at Day 1 and 3, the decrease in total IRS-1 protein at Day 3 leads to an increase in the amount of p85 relative to IRS-1 (Figure 3C). IRS-2 associated p85 decreased from subconfluent levels reaching significance on Day 1 (subconfluent = 0.96 ± 0.05, Day 1 = 0.56 ± 0.08) and remained suppressed up to Day 3 (0.56 ± 0.05; Figure 3D). Therefore, reaching confluence reduces signaling through IRS-1, while IRS-2 signaling reduces following serum withdrawal.

### Knockdown of S6K1 does not alter IRS-1 levels or differentiation

**Figure 4:**
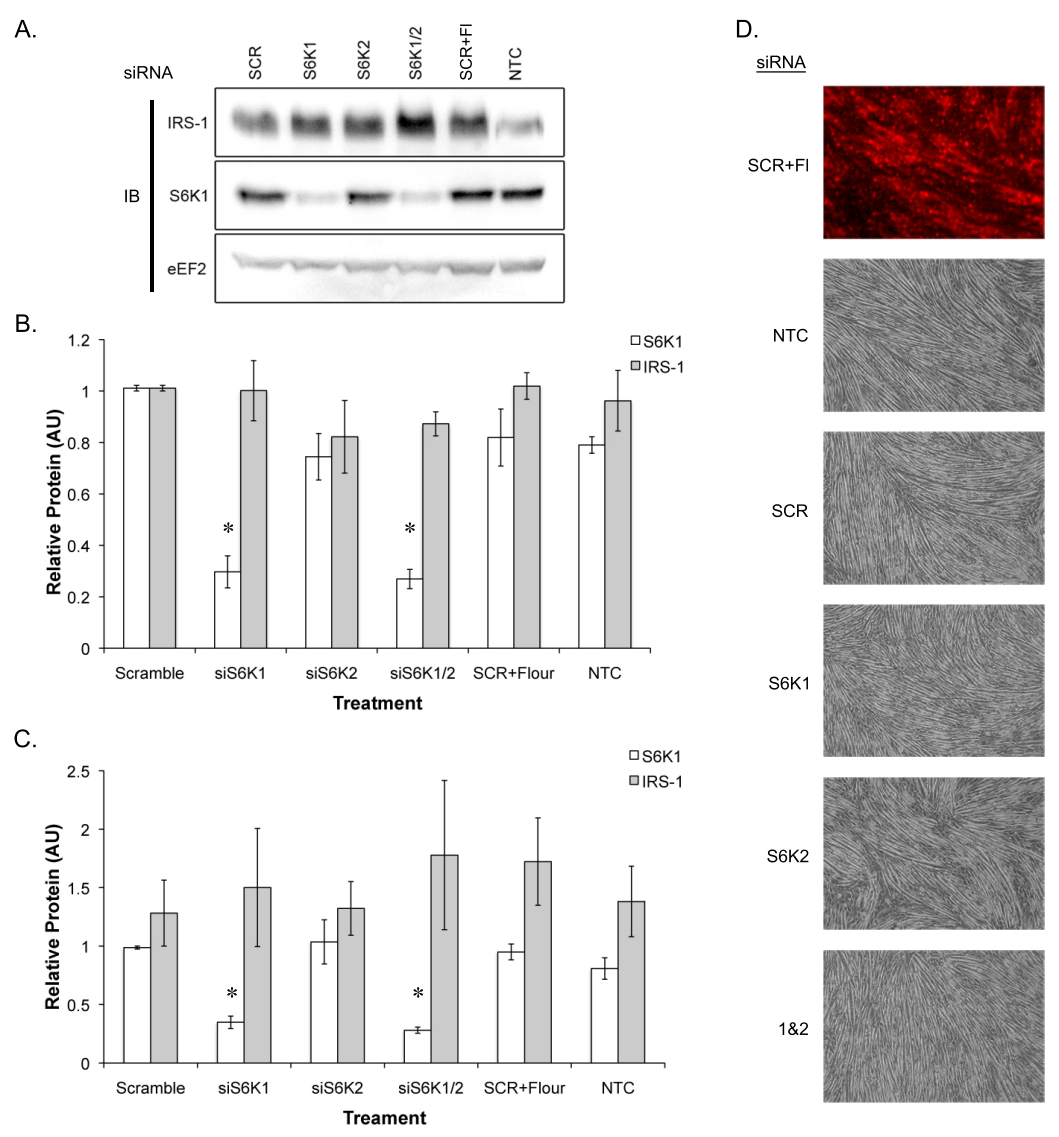
**S6K1 siRNA knockdown does not affect IRS-1 protein or muscle cell differentiation**. A) Representative Blots, B) IRS-1 and S6K1 expression following treatment with siRNA directed against S6K1 and S6K2 for 48hrs post transfection (cells transfected at SC and collected at Day 1), C) IRS-1 and S6K1 levels relative to eEF2 following treatment with siRNA directed against S6K1 and S6K2 for 96 hrs (cells transfected at SC and collected at Day 3), D) Representative images of myotube formation following 96hrs siRNA treatment. Figure headings - SCR = scramble control siRNA, S6K1 = siRNA against S6K1, S6K2 = siRNA against S6K2, S6K1/2 = siRNA against both S6K1&2, SCR+Fl = scramble control plus a fluorescent tagged siRNA, NTC = no treatment control. * indicates significantly differently different from all other treatments.

To determine whether increasing S6K1 activity underlies the decrease in IRS-1 protein and activity, siRNA directed against S6K1 and its homologue S6K2 were transfected into muscle cells prior to differentiation. Transfection with siS6K1 for 48 hrs (transfected at SC, collected at Day 1) significantly reduced S6K1 protein levels (Figure 4A). However, despite the robust knockdown of S6K1 there was no effect on IRS-1 protein levels (Figure 4B). To determine whether loss of S6K1 was compensated for by its homologue S6K2, we knocked down S6K2 individually or in combination with S6K1 and measured the effects on IRS-1 levels and differentiation. Knock down of S6K2 or S6K1 and 2 had no effect on IRS-1 protein levels (Figure 4B) or myotube formation (data not shown). Since the decrease in IRS-1 protein was not apparent until Day 3, the effects of a more sustained knockdown (collecting the cells at Day 3) on IRS-1 levels and differentiation were determined. The 96 hr knockdown yielded similar results: no increase in IRS-1 protein levels (Figure 4C) and no change in myotube formation (Figure 4D). These data suggest that the physiological activation of S6K1 does not mediate the decrease of IRS-1 protein level during myoblast differentiation and has no effect on myotube formation.

### Differentiation-induced phosphotyrosine associated p85

**Figure 5:**
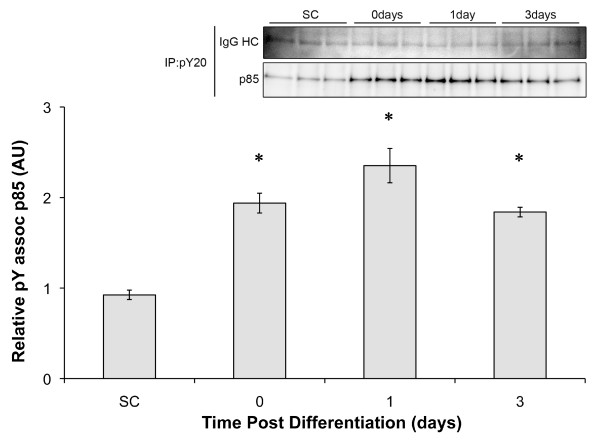
**Differentiation increases phosphotyrosine associated p85**. Representative western blot of phosphotyrosine associated p85 in C2C12 cells at 3 (subconfluent; SC), 24 (Day 0), 48 (Day 1), or 96 hours (Day 3) following plating. Phosphotyrosine complexes were immunoprecipitated and the associated p85 was determined by western blot and quantified using the IgG HC as a control. * indicates significantly higher than in subconfluent cells.

Since PI3K signaling is known to be required for myotube formation, yet IRS-1/2 associated p85 was decreased, phospho-tyrosine associated p85 was determined to assess non-IRS associated PI3K activation. pY associated p85 increased approximately 2-fold (SC = 0.92 ± 0.05, Day 0 = 1.93 ± 0.11; Figure 5) upon reaching confluence and remained significantly elevated throughout the 72 hrs of the experiment (Day 3 = 1.84 ± 0.05). These data suggest that PI3K is activated within the myoblasts upon reaching confluence and that the predominant route for PI3K signaling is through non-IRS-1/2 phospho-tyrosine complexes.

## Discussion

In C2C12 cells, S6K1 activity increased almost 3-fold from subconfluence until myotubes were completely formed. Concomitant with the increase in S6K1 activity was a decrease in IRS-1 signaling and protein levels, consistent with the negative feedback loop from S6K1 to IRS-1. However, knocking down S6K1 had no effect on IRS-1 protein levels, suggesting that physiological activation of S6K1 during differentiation did not lead to the reduction of IRS-1 protein. Even though IRS-1/2 function decreases during differentiation, phosphotyrosine associated p85 increases at confluence and remains high throughout differentiation. These data suggest that during myogenesis the predominant source of PI3K activity is phosphotyrosine complexes other than IRS-1/2.

S6K1 activity increases substantially from subconfluent C2C12 myoblasts to fully differentiated myotubes. Recently, S6K1 has been identified as a key component of a negative feedback loop in insulin/growth factor signaling [19]. This feedback is mediated through serine phosphorylation and direct regulation of IRS-1 protein and mRNA. In differentiated L6 myotubes, activation of S6K1 for as little as 1hr, inhibits insulin stimulated IRS-1 associated PI3K activity [18]. Therefore, we hypothesized that the prolonged activation of S6K1 during myogenesis would lead to a reduction in IRS-1 protein levels. Consistent with this hypothesis, IRS-1 protein declined by more than 50% between Day 1 and Day 3 of differentiation. Since growth factor mediated differentiation is thought to involve IGF-II signaling through IRS-1 to PI3K and PKB, we further hypothesized that decreasing S6K1 would increase IRS-1 protein and result in greater IGF-II signaling and myoblast differentiation. Contrary to this hypothesis, knockdown of S6K1, its structural homologue S6K2, or both enzymes together had no effect on IRS-1 protein level or muscle cell differentiation. These data suggest that during differentiation S6K1 does not regulate the level of IRS-1 protein or muscle cell differentiation. In agreement with the current work, Park et al [16,20] have elegantly shown that S6K1 is not required for muscle cell differentiation but plays an important role in myocyte hypertrophy once differentiation is complete.

Measuring the amount of p85 associated with a particular scaffolding protein provides an estimate of the PI3K activity of that complex. Due to the requirement on IRS-1 for human myoblast differentiation [21] we had expected that IRS-1 associated p85 would be substantially elevated from the point of plating. However, both IRS-1 and IRS-2 associated p85 were significantly suppressed during differentiation. Instead of associating with IRS-1 or 2, PI3K associates with other phosphotyrosine complexes during differentiation in C2C12 cells. These data do not preclude a role for IRS in myogenesis. Indeed, cross-talk between cell surface receptors and the insulin/IGF-I pathways has recently been described in mice with skeletal muscle lacking β_1_-integrins [22]. In mice without β_1_-integrins insulin-induced phosphorylation of PKB at Ser473 was impaired even though IRS-1 signaling appeared normal. Since integrins and cadherins interact with integrin-linked kinase (ILK;[23,24], and ILK binds to both PKB and the Ser473 kinase mTORC2 [25], this suggests that cell surface receptors work together with IRS-1 to activate PI3K and PKB. The current data suggest that in differentiating muscle, the interaction between the cell surface receptors and IRS-1 is reversed. At high confluence, cell-cell contact results in the activation of cadherins and integrins [26], which function as high confluence-activated receptors. PI3K can be recruited to cell-cell contacts and activated by cadherin [27], but this has yet to be demonstrated in muscle cells. During differentiation, cadherins are upregulated in C2C12 cells [28], suggesting that they may play an important role in confluence-dependent differentiation. Some researchers have suggested that cadherins can activate PKB during differentiation, but this has previously been attributed to Rho kinase [4] or Cdo/JLP [5] dependent activation of p38. The current data suggest a more direct mode of activation is possible: the direct recruitment of PI3K, possibly in association with a permissive level of activated IRS-1, to the membrane and the local production of PIP_3_. While the cadherins have yet to be definitively identified as the phospho-tyrosine containing protein that increases its interaction with p85 during differentiation, the location, function, and dynamics of cadherins during differentiation are all suggestive of a role for these proteins.

Our profile of PKB phosphorylation differs from that previously published [29,30]. We observed a biphasic response, whereas others observe a linear increase in PKB phosphorylation at Ser473 and Thr308 [29,30]. This discrepancy could be due to the differentiation protocol used. We include data from cells at subconfluence and withdraw serum at 90-100% confluence whilst Deldicque et al [29] withdrew serum at 70% confluence. Gonzalez et al. [31] include data from cells at subconfluence however they collected their cells at a higher confluence and they plated their cells on gelatin coated plates which could affect the cell surface receptors that may be involved in PKB activation. These differences may explain why Gonzalez et al. did not observe an initial reduction in PKB Thr308 phosphorylation upon reaching confluence. Using a similar protocol and measuring PKB activity directly, Cuenda and Cohen observed the same increase in PKB activity 1.5 days following serum withdrawal followed by a decrease at day 3 [15]. These results highlight the importance of maintaining a consistent protocol for differentiation studies.

## Conclusions

Reducing S6K1 protein in differentiating myoblasts does not prevent the loss of IRS-1 at Day 3 of differentiation or improve muscle cell differentiation, suggesting that there are physiological situations when S6K1 is activated and yet IRS-1 is not adversely affected. Furthermore, IRS-1/2 do not appear to contribute substantially to overall PI3K activity during muscle cell differentiation, suggesting that other, as yet undetermined proteins, mediate this essential aspect of muscle cell differentiation.

## Methods

### Materials

All plasticware for tissue culture was from Greiner (VWR, UK). Tissue culture media and sera were from Invitrogen. Anti-IRS-1 and anti-IRS-2 antibodies were obtained from the Division of Signal Transduction Therapy (DSTT; University of Dundee). pY20 was obtained from BD Biosystems (New Jersey, USA). Total myosin heavy chain (MF20) and the myogenin (F5D) antibodies were obtained from the Developmental Studies Hybridoma Bank developed under the auspices of the NICHD and maintained by The University of Iowa, Department of Biological Sciences, Iowa City, IA 52242. Antibodies for p-PKB^T308^, t-eEF2, S6K^T389^, p-p44/42^Thr202/Tyr204^, t-p44/42, and t-PKB were from Cell Signaling Technologies and were used at 1:1000. The total S6K antibody was from Santa Cruz Biotechnologies and was used at 1:1000. Secondary antibodies were from Perbio Science (Cramlington, UK) and used at 1:10000. All phospho-specfic antibodies were quantified relative to their total protein, whereas IRS-1/2 and MHC were quantified relative to eEF2. Total eEF2 protein content was unchanged by any of the treatment or interventions. Thr308 phosphorylation was used to measure the effects of differentiation of PKB since this is the primary PI(3,4,5)P_3_-dependent site [32] and therefore the most directly regulated by IRS-1/2. Chemicals were from Sigma-Aldrich unless stated otherwise.

### Tissue culture and transfection

C2C12 myoblasts were grown in growth media [Dulbecco's Modified Eagle's Medium (DMEM) supplemented with 10% fetal bovine serum (FBS) and 1% penicillin/streptomycin]. Myoblasts were kept below 60% confluence to prevent confluence-induced differentiation. Cells for immunohistochemistry were plated at approximately 200000 cells/well in 6-well plates on collagen coated coverslips and fixed after 3 hrs (subconfluent) or at confluence (24 hr; time Day 0). On the remaining cells, the media was changed to differentiation media (DMEM supplemented with 2% HS and 1% penicillin/streptomycin). Cells were then fixed 24 hrs (Day 1) or 72 hrs (Day 3) following differentiation. Cells for immunoprecipitations, kinase assays and western blots were plated on 10 cm plates at 1.2 million cells per plate and differentiated as above. Each experiment was performed on three separate occasions with n = 4 for each experiment. Data presented is representative data from a single experiment.

### RNA interference of S6K1 and S6K2

Specific siRNA was designed using the siGENOME *SMART*pool approach (Dharmocon RNAi Techonlogies, Thermo Scientific, UK) based on the mouse sequence for S6K1 (NM_028259) and S6K2 (NM_021485). Prior to transfection with siRNA, cells were seeded on 12 well plates at 80000 cells per well with the transfection carried out 3 hours following seeding. Cells were washed × 3 with sterile PBS and 1 ml serum and antibiotic free DMEM was added to the cells prior to the addition of transfection medium. The transfection medium consisted of 200 nmol of siRNA and an equal amount (1:1) of lipofectamine 2000 reagent suspended in optimem transfection media (Invitrogen, UK). siRNA was conjugated with lipofectamine for 30 min before being added to the cells for 18 h. At the end of 18 hours, cells were washed × 3 with PBS and moved to differentiation media. Mouse ON-TARGET*plus *Non-targeting siRNA (# D-001810-10, Dharmacon RNAi technologies) was used as a control during all experiments, whilst siGLO (Dharmacon RNAi technologies) was used to assess primary and secondary transfection efficiency.

### Protein extraction and SDS-PAGE

At the allocated time points, cells were washed with ice cold PBS and lysed in sucrose lysis buffer (50 mM Tris pH7.5, 250 mM Sucrose, 1 mM EDTA, 1 mM EGTA, 1% Triton × 100, 50 mM NaF, 1 mM NaVO_4 _Na_2_(PO_4_)_2 _and 0.1% DTT). The lysate was briefly vortexed and centrifuged at 4°C for 10 mins at 11000 rpm to remove insoluble material. Protein concentrations were determined using the DC protein assay (Bio-Rad, Hercules CA). Equal aliquots of protein were diluted in Laemmli sample buffer and boiled for 5 mins. 5-10 μg of sample was then subjected to SDS-PAGE on 10% acrylamide gels and transferred to Protran nitrocellulose membrane (Whatman, Dassel, Germany) and blocked in 5% dry milk in TBS-T.

### Immunobloting

Membranes were incubated in phosphospecific antibodies overnight at 4°C, washed and then incubated in HRP conjugated secondaries at 1:10000 for 1 hr at room temperature. Following a final wash, the membranes were incubated in Immobilon™ Western Chemiluminescent HRP Substrate (Millipore, Billerica, MA) and exposed in a Chemigenius Bio-imaging System (Syngene, Cambridge, UK). The membranes were then stripped in Restore™ Western Blot Stripping Buffer (Pierce, Rockford, IL) for 20 mins and reprobed for total protein content. All phosphorylation specific antibodies were normalised to the relevant total protein antibody when available, or to eEF2 when this was not possible.

### S6K1 activity assay

S6K1 activity was measured as previously described [33]. Briefly, endogenous S6K1 was immunoprecipitated from1 mg total protein for 2 hours at 4°C with 1 μg of rabbit anti-S6K1. S6K1 protein was then immobilised on protein-G sepharose for one hour. Immunocomplexes were washed one time in high detergent buffer A (1% NP-40, 100 mM NaCl, 10 mM Tris pH 7.2, 1 mM EDTA), two times in high salt buffer B (1M NaCl, 0.1% NP-40, 10 mM Tris pH 7.2), and one time in ST buffer (150 mM NaCl, 50 mM Tris pH 7.2). The immunocomplexes were resuspended in 20 μl of 1.5 × kinase buffer (200 mM Hepes pH 7.2, 100 mM MgCl_2_, 1 mg/ml BSA. 1:3000 2-Mercaptoethanol) and the activity towards a recombinant GST-S6 peptide was assayed for 10 mins at 30°C. Reactions were initiated and terminated using 10 μl ATP mix (50 μM unlabelled ATP, 5 μCi of [γ-^32^P] ATP, 1 μl GST-S6) and 4 × LSB (0.5 M Tris pH 6.8, 0.8% SDS, 20% 2-Mercaptoethanol, 30% Glycerol), respectively. Reactions were subjected to 10% SDS-PAGE and incorporation of ^32^P into GST-S6 was assessed by autoradiography and quantified using a Chemi Genius Bioimaging Gel Doc System (Syngene, Cambridge, UK).

### Immunocytochemistry

Coverslips containing cells (see above) were rinsed twice in room temperature PBS before being fixed in 4% paraformaldehyde for 20 mins. Coverslips were then rinsed 3 times in PBS and aldehydes were quenched in 100 mM glycine for 15 mins before rinsing 3 times in 0.2% fish skin gelatine (made in PBS). Coverslips were then blocked in Goat Serum (1:10, 0.2% FSG; Jackson ImmunoResearch Europe) for 10 mins, and rinsed once in 0.2% FSG before being incubated in primary antibodies [MyoD1 (Novocastra Laboratories) prepared according to the manufacturer's instructions at 1:50 or Myogenin/F5D at 1:50] for 20 mins at room temperature. Primary was rinsed 3 times with 0.2% FSG before being placed in secondary [Alexa Fluor 488 (Invitrogen) at 1:500, with DAPI at 1:1000 using an 10 μg/μl stock]. Secondary was rinsed off 3 times with 0.2% FSG and 3 times in MiliQ water before being blotted dry and mounted in Vectashield^® ^Mounting Medium (Vector Laboratories). Coverslips were then analyzed on a fluorescent microscope. To analyze MyoD and Myogenin expression, 5 different fields were counted for DAPI positive nuclei and MyoD/Myogenin positive nuclei.

### Statistics

All data are represented as mean ± standard error and significance was determined using the Brightstat statistical package http://www.brightstat.com using ANOVA with posthoc analysis by Tukey's Honestly Significant Difference test.

## Authors' contributions

DLH carried out the cell experiments, pulldowns and blots and drafted the manuscript. AP assisted with cell culture, carried out some of the immunoblots and helped to draft the manuscript. MGM carried out the S6 K assays. KB conceived the study, and participated in its design and coordination and helped to draft the manuscript. All authors read and approved the final manuscript.
